# Community attitudes and Indigenous health disparities: evidence from Australia's Voice referendum

**DOI:** 10.1016/j.lanwpc.2024.101154

**Published:** 2024-08-01

**Authors:** Karinna Saxby, Zoe Aitken, Luke Burchill, Yuting Zhang

**Affiliations:** a Melbourne Institute: Applied Economic & Social Research, Faculty of Business and Economics, University of Melbourne, Melbourne, Victoria, Australia; b Centre for Health Policy, Melbourne School of Population and Global Health, The University of Melbourne, Melbourne, Victoria, Australia; c Royal Melbourne Hospital, The University of Melbourne, Melbourne, Victoria, Australia; d Department of Cardiovascular Medicine, Mayo Clinic, Rochester, MN, USA

**Keywords:** Indigenous, Health disparity, Community attitudes, Structural racism, Australia, Risky behaviour, Healthcare, Mental health, Discrimination

## Abstract

**Background:**

Community attitudes influence health outcomes especially for racially diverse and minority groups exposed to the detrimental effects of racism and discrimination. Using the results from Australia's national referendum to establish an Aboriginal and Torres Strait Islander Voice to Parliament (‘the Voice’) as a proxy for attitudes to Indigenous Australians, this study examined health outcomes for Indigenous and non-Indigenous Australians according to levels of opposition to the Voice.

**Methods:**

The regional share of votes against the Voice was linked to 2021 data from the Household, Income and Labour Dynamics in Australia survey, a large, national probability sample (n∽17,000) of Australian adults. Adjusting for regional-level confounders, we used logistic regression analyses to predict health outcomes, healthcare use, and risk-taking behaviours among Indigenous and non-Indigenous Australians for different levels (quartiles) of opposition to the Voice.

**Findings:**

Greater opposition to the Voice was associated with widening Indigenous disparities in health, healthcare use, and health behaviours. Indigenous Australians living in regions with the highest opposition to the Voice (top quartile: ≥72% community voting ‘No’) were more likely to report fair/poor health [OR 2.28 (95% CI 1.45–3.58)] and poor mental health [OR 2.24 (95% CI 1.48–3.39)], were less likely to have visited any healthcare provider [OR 0.52 (95% CI 0.36–0.75)], and were more likely to smoke [OR 4.21 (95% CI 2.78–6.38)] or engage in risky drinking [OR 2.66 (95% CI 1.60–4.43)] relative to non-Indigenous Australians.

**Interpretation:**

Indigenous Australians living in communities with greater opposition to the Voice experience poorer health relative to non-Indigenous Australians. Disparities in health may be partially due to poorer healthcare access and increased risk-taking behaviours, which may be associated with racism. These findings align with discrimination-related stress processes and potentially reduced availability of culturally inclusive healthcare. Health and social policy should consider how broader societal level conditions shape Indigenous health disparities in Australia.

**Funding:**

This work is supported by the 10.13039/501100000923Australian Research Council (project ID FT200100630), the University of Melbourne Faculty Research Grant, and the 10.13039/501100000925National Health and Medical Research Council of Australia Investigator Grant (project ID 1201937).

**Research in context**

**Evidence before this study**

Racism is associated with negative health outcomes among Aboriginal and Torres Strait Islander ‘Indigenous’ peoples. Previous studies have demonstrated that structural factors and societal level conditions, including community-level attitudes, can shape experiences of interpersonal discrimination among racial and minority groups. While the detrimental health effects of racism at the interpersonal level is well documented, less is known regarding the community-level impacts of negative societal attitudes on Indigenous health behaviours and outcomes.

**Added value of this study**

We show that Indigenous populations living in regions with high opposition to the Voice experience poorer health outcomes. Potential factors contributing to these disparities include reduced healthcare access and increased risk-taking behaviours among Indigenous Australians living in areas with high opposition to the Voice. Conversely, opposition to the Voice is not associated with these same outcomes for non-Indigenous Australians.

**Implications of all the available evidence**

Community-level opposition to the Voice is associated with poorer health outcomes for Indigenous, but not non-Indigenous, Australians. Internalisation of negative attitudes and experiences of discrimination may lead to reduced healthcare access and higher risk-taking behaviours, which themselves are adversely influenced by exposure to racism. Multi-level reforms supporting culturally safe communities and healthcare pathways for Indigenous Australians are needed to address structural discrimination and reduce Indigenous health disparities.

## Introduction

Indigenous health disparities are a pressing global concern. A growing body of empirical evidence suggests that structural discrimination—societal level conditions which foster racial discrimination^1^—can influence racial disparities in health outcomes through exposure to physical and psychological stressors, inducing risk-taking behaviours, and reduced engagement with preventative healthcare.^2^, ^3^, ^4^, ^5^, ^6^ There are currently no universal approaches to measure structural discrimination. However, societal-level attitudes towards minority groups are increasingly being used to conceptualise structural discrimination and capture minority stressors as they can not only reflect broader institutional factors, such as laws and policies, but also influence them.^7^^,^^8^

While previous research has documented negative health effects of interpersonal racism among Indigenous populations,^9^, ^10^, ^11^ to our knowledge, no studies have investigated how community-level attitudes influence Indigenous health outcomes or disparities relative to non-Indigenous peoples. This is a critical research gap when considering the impact of colonisation on Indigenous vs non-Indigenous people and the explicit and tacit ways in which systems of racial oppression act to limit the human rights, health, and wellbeing of Indigenous peoples.^1^ Recognising the harms of colonisation are deep and continue for Indigenous peoples, and given Indigenous communities themselves are diverse and everchanging, it is critical that Indigenous health outcomes be examined with attention to the contemporary sociopolitical and structural factors which shape Indigenous lives and outcomes today.^1^^,^^12^ Country-specific research on structural factors is therefore urgently needed.

The scant research in this space is partially because aggregated measures of community attitudes or beliefs which may reinforce social exclusion of Indigenous peoples are difficult to quantify. While community surveys have been used to investigate predictors of negative attitudes towards Indigenous populations,^13^^,^^14^ these estimates are likely to be biased by social desirability and sample selection.^13^ Proxying community attitudes through aggregate measures of interpersonal racism are also imprecise as experiences of racism are known to be underreported.^10^^,^^15^

In this paper, we address this crucial knowledge gap by utilising the results from the 2023 Australian referendum on altering the Constitution to recognise an Aboriginal and Torres Strait Islander Voice in Parliament (hereafter ‘the Voice’) as a proxy for community attitudes towards Indigenous Australians. We examine the extent to which regional opposition to the Voice influences Indigenous health disparities in Australia.

This study thus expands upon emerging evidence that community-level opposition to the Voice may capture negative attitudes towards Indigenous Australians^16^ albeit leveraging broader hypotheses from the structural discrimination and health literature. Specifically, we hypothesise that should community-level opposition to Indigenous peoples having a political Voice reflect structural discrimination, higher opposition to the Voice would be associated with poorer health outcomes for Aboriginal and Torres Strait Islander ‘Indigenous’ peoples, but not for non-Indigenous Australians, resulting in larger Indigenous health disparities in areas of high opposition.

## Methods

### Engagement with Aboriginal and Torres Strait Islander peoples

Engagement with Aboriginal and Torres Strait Islander peoples is central to this research and responds to longstanding priorities as endorsed by the Aboriginal and Torres Strait Islander community. Details on research alignment to these priorities as well as engagement with Aboriginal and Torres Strait Islander peoples throughout the research process is provided in Supplementary Material SM1.

### Data

We use 2021 data (most recently available at the time of analyses) from the Household, Income and Labour Dynamics in Australia (HILDA) survey. HILDA is an annual survey of over 17,000 Australians, with data currently available from 2001 (wave 1) to 2021 (wave 21).^17^ The HILDA Survey uses a complex probabilistic sampling design and has high retention rates.^17^ While HILDA is largely representative of Australians aged 15 and older, it does not include those living in very remote areas, who are more likely to be Indigenous. However, the majority of Indigenous Australians live in urban and regional centres. A comparison of Indigenous peoples in the 2021 HILDA sample to those in the 2021 Census (Supplementary Material SM2) shows that Indigenous peoples in the HILDA sample are, on average, older, less likely to reside in rural or socioeconomically disadvantaged areas, and have higher levels of education than Indigenous peoples in the Census.

Wave 21 of HILDA (i.e., carried out in 2021) is useful for our analysis as it contains not only information on individuals’ Indigenous status, residential location, and subjective health measures (which are measured every-wave), but additionally includes specific questions on health care use and risk-taking behaviours (which are only collected every four years). Information on Indigenous status is based on responses to the question “Are you of Aboriginal or Torres Strait Islander origin” with the following response options: “Not of indigenous origin”, “Aboriginal”, “Torres Strait Islander,” or “Both Aboriginal and Torres Strait Islander.” We define Indigenous peoples as those who identify as Aboriginal, Torres Strait Islander, or both.

### Outcome measures

Following previous research on the health effects of discrimination,^2^^,^^10^ we focus on self-reported self-reported health outcomes: fair/poor general health, poor mental health, and disability. Fair/poor general health is based on the question “How is your health in general? Would you say it was excellent, very good, good, fair, or poor?” We create a binary variable, identifying those in fair/poor general health. Poor mental health is measured using the mental health subscale of the SF-36, also known as the Mental Health Inventory, or MHI-5.^18^ The index is an additive index ranging from zero (worst mental health) to 100 (best mental health) and is constructed using five questions about how much of the time in the past four weeks respondents had been nervous, felt calm and peaceful, felt down, and felt happy. Individuals are classified as being in poor mental health if they have a score less than 50.^18^ Disability is defined as individuals reporting having an “impairment, long-term health condition or disability which restricts their everyday activities that had lasted, or was likely to last, for a period of 6 months or more.”

We next consider healthcare use and risk-taking behaviours as potential mechanisms through which societal-level conditions could impact health. For example, individuals may internalise negative attitudes, believe they are ‘less worthy’ and in turn deprioritize their health and self-care.^2^^,^^4^ People may use alcohol or other drugs to cope with discrimination-related stressors. Broader societal-level conditions and attitudes could also influence the availability and provision of culturally inclusive healthcare, and at the same time, reduced engagement with healthcare could reflect preexisting experiences of discrimination within the healthcare system. To explore this, we look at three measures of healthcare use in the previous 12 months: whether individuals have seen any healthcare provider; used any prescription medications; and any reported hospital care. Last, we examine risk-taking behaviours as potential measures of reduced self-care; namely, tobacco use smoking, alcohol consumption, and drug use. Current and ex-smokers are defined based on response to the questions, “Do you smoke cigarettes or any other tobacco products?”; i.e., if they responded “No, I have given up smoking,” and “Yes.” Next, individuals are classified as engaging in risky drinking if they exceeded the sex-based threshold on one occasion (5+ standard drinks for women and 7+ for men) “2 or 3 times a month”, “1 or 2 times a week,” “3 or 4 times a week,” or “5 or more times a week” within the past year.^19^ Illicit drug use is based on responses to question “in the last 12 months, how often did you use each of the following types of drugs?” with the following options: “marijuana/cannabis,” “meth/amphetamine,” “cocaine,” “ecstasy,” “hallucinogens,” and “any other illicit drug.” To each drug type, individuals could respond “Every day,” “Once a week or more,” “2 or 3 times a month,” “About once a month,” “Every few months,” “Once or twice a year,” or “Not at all.” A binary variable equal to 1 is created if the individual recorded anything other than “Not at all” for any of these illicit drugs.

### Community attitudes—the Voice referendum

In October 2023, Australia held a referendum about whether to change the Constitution to explicitly recognise the First Peoples of Australia by establishing an advisory body—an Aboriginal and Torres Strait Islander Voice—to Parliament and the executive Government. Aboriginal and Torres Strait Islander peoples having a voice in parliament was part of a continuing effort to advance Indigenous self-determination in Australia.^12^ Specifically, the referendum honoured the Australian Government's commitment to implement the 2017 Uluru Statement from the Heart, which involved extensive and nationwide consultation with Indigenous Australians and codesign processes with communities and both Indigenous and non-Indigenous organisations.^12^

Coined the Voice referendum, all eligible voting Australians were mandated to participate and were posed the following question, with a “yes” or “no” response: “A Proposed Law: to alter the Constitution to recognise the First Peoples of Australia by establishing an Aboriginal and Torres Strait Islander Voice. Do you approve this proposed alteration?”^20^ The results showed that approximately 61% of Australians voted against the Voice. Analyses of voting responses suggest that those voting ‘no’ were more likely to be male, have lower levels of education, and lower household incomes.^16^ There is also evidence that Aboriginal and Torres Strait Islander peoples were more likely to vote yes than non-Indigenous Australians.

**Fig. 1:**
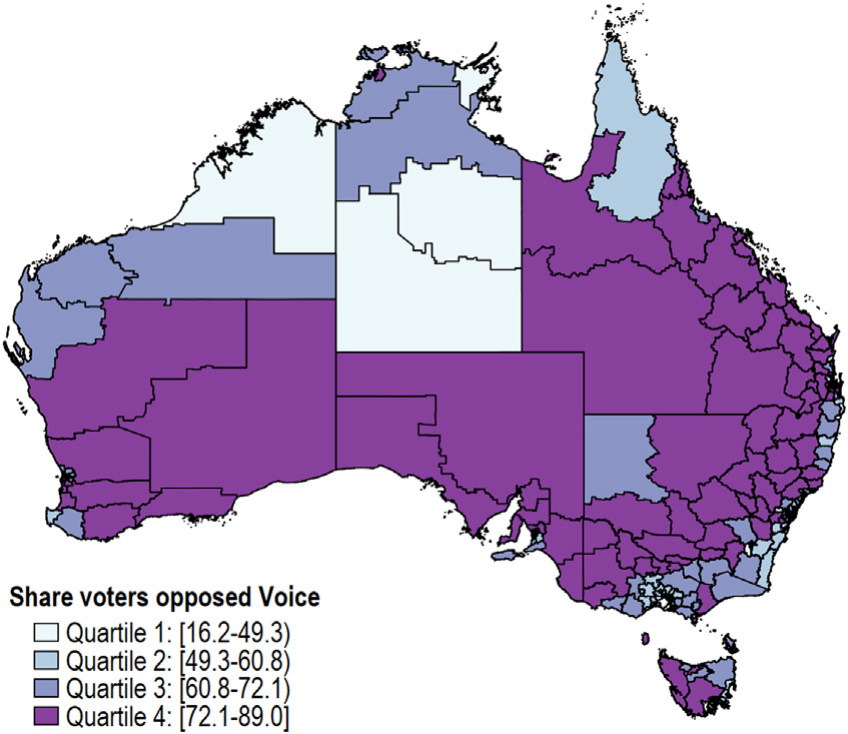
**Geographical variation in share of voters against embedding an Aboriginal and Torres Strait Islander Voice into the Constitution, Australia. Notes: Based on responses to the 2023 Referendum on an Aboriginal and Torres Strait Islander Voice at the Statistical Area 3 Level**.

There was substantial regional variation in the responses to the referendum (Fig. 1). Across different Statistical Area-3 (SA3) regions (which have populations ranging between 30,000 and 130,000 people), the share of votes against the Voice had a coefficient of variation of 0.25 (mean/standard deviation, with larger numbers indicating more variability to the population mean) and ranged from 16% to 89%. We use SA3-regions as they closely represent suburbs and have similar geographic and socioeconomic characteristics. We exploit this regional variation to identify whether higher opposition to the Voice is associated with disparities among Indigenous Australians. Specifically, we categorise the percentage of votes against the Voice out of those eligible to vote in each SA3-region into quartiles: Q1 [16.2–49.3%); Q2 [49.3–60.8%); Q3 [60.8–72.1%); and Q4 [72.1–89.0%].

### Statistical analyses

For all individuals in our sample, we first assign their regional level of opposition to the Voice based on their SA3 of residence at the time of the 2021 HILDA Survey. We then apply logistic regression models for each outcome pertaining to reporting poor health (fair/poor general health; poor mental health; disability); any healthcare use (seen any healthcare provider; used any prescription medications; any hospital care); and risk-taking behaviour (smoking; risky drinking; illicit drug use).

We estimate each model by including an interaction term between the quartile of opposition to the Voice and Indigenous status as an explanatory variable, and including HILDA sample probability weights.^17^

Selection of potential confounders is an important consideration in this specification and, to this end, we closely follow approaches employed by similar studies in the Australian setting.^3^^,^^10^^,^^21^ Under the scenario in which structural discrimination did not exist, there should be no systemic differences in health outcomes, healthcare use, and risk-taking behaviours between Indigenous and non-Indigenous Australians.^10^ While there is an argument that geographical segregation could disproportionately affect access to resources, selection itself into regions could be a result of structural discrimination.^3^ As we cannot formally test this in our data, we instead opt to explore how community-level attitudes are associated with health inequalities, even when matching regions to be similar on observable characteristics which are known to affect healthcare access. Specifically, we control for place-based factors which could impact health including area-level disadvantage, population density, and state/territory fixed effects.

We additionally control for individual-level age categories (‘15–39 years,’ ‘40–59 years,’ ‘60 plus years’) and sex (‘male’, ‘female’). As in previous studies, we do not control for other potential individual-level confounders which may impact outcomes, such as employment or education, as denial of access to goods and services, including employment and vocational opportunities, are mechanisms through which structural discrimination could operate.^10^ Nevertheless, as robustness checks, we explore the robustness of our results when controlling for age as a continuous variable and including additional individual-and unobservable regional-level factors (educational attainment, labour force participation, marital status, children in household, equivalised household income, and SA3-fixed effects).

Disparities in outcomes between Indigenous and non-Indigenous people are calculated for each quartile of opposition to the Voice as odds ratios by combining coefficients for the Indigenous indicator and the interaction term for each quartile of opposition from the logistic regression model. Full details of the model specification are provided in Supplementary Material SM3 and we report the full results from these models as odds ratios (OR) with 95% confidence intervals in Supplementary Material SM4. We then compute adjusted average predicted probabilities of the outcomes, and their corresponding Wald 95% confidence intervals, for non-Indigenous and Indigenous Australians in each quartile of opposition to the Voice at the observed values of the covariates in the model.^22^ Presenting differences across quartiles of opposition in this way allows us to readily test for non-linear associations,^3^^,^^21^ facilitate tangible interpretation of results, and identify whether disparities are attributable to differences in the prevalence of outcomes across quartiles of opposition for Indigenous or non-Indigenous Australians. We rescale all predicted probabilities from 0 to 1 to percentages (0–100) for readability. Lastly, we perform Wald tests to assess whether disparities in Q2-Q4 are statistically different to Q1. The full results and the associated hypothesis testing are provided in Supplementary Material SM5. All analyses are conducted using STATA version 17. The study was approved by the Office of Research Ethics and Integrity at the University of Melbourne.

### Ethics committee approval

The Office of Research Ethics and Integrity at the University of Melbourne has approved this study (Project ID 29421). Consent for participation in HILDA is obtained verbally when the respondent agrees to be interviewed. For persons, 15–18 years old living at home, interviewers obtain verbal permission from their parent. This information and associated ethical approval statement for Wave 21 of HILDA has been approved by The Office of Research Ethics and Integrity at the University of Melbourne (Project ID 13551).

### Role of the funding source

Funding bodies had no role in study design, data collection, data analysis, data interpretation, or writing of the report. The authors had final responsibility for the decision to submit for publication, once approved by Ethics Committees.

## Results

### Descriptive statistics

The descriptive characteristics of the study sample are presented in Table 1. Our dataset comprises 15,993 non-Indigenous Australians and 544 Indigenous peoples. Compared to non-Indigenous Australians, on average, Indigenous peoples were younger (mean 35.9 vs. 46.7 years), had lower levels of educational attainment, and higher rates of unemployment. Indigenous peoples in the sample were also more likely to live in areas with higher socioeconomic disadvantage, lower population density, and higher opposition to the Voice (mean 65.8 vs 58.5%). Compared to non-Indigenous Australians, a higher proportion of Indigenous peoples reported being in poor mental health (26 vs 14%), fair or poor general health (23 vs 17%) and a disability (28 vs 23%) but Indigenous peoples were less likely to have seen any healthcare professional (57 vs 71%), taken prescription drugs (57 vs 71%) in the past 12 months. Risk-taking behaviours were slightly higher among Indigenous Australians relative to non-Indigenous Australians. Indigenous peoples were more likely to smoke or be ex-smokers (66 vs 43%), have engaged in risky drinking (30 vs 20%), and have used illicit drugs (51 vs 43%).

**Table 1:** Descriptive statistics.

	Non-Indigenous (n = 15,993)		Indigenous (n = 544)	
	Mean (SD)/Prop.	Freq.	Mean (SD)/Prop.	Freq.
**Individual characteristics**				
Age	46.7 (19.31)	–	35.9 (15.89)	–
Age group				
15–39 years	0.42	6706	0.67	363
40–59 years	0.30	4732	0.22	118
≥60 years	0.28	4555	0.12	63
Male	0.47	7527	0.44	238
Educational attainment				
Less than High school	0.22	3548	0.38	208
High school or equivalent	0.38	6062	0.45	247
Bachelor or above	0.40	6377	0.16	87
Labour force status				
Employed	0.64	10,227	0.54	292
Unemployed	0.03	490	0.10	56
Not in the labour force	0.33	5276	0.36	196
**Regional characteristics**				
Population density (/m2)	1131 (1453)	–	641 (1216)	–
Major cities	0.69	10,980	0.47	253
Inner regional	0.22	3556	0.35	187
Outer regional	0.08	1318	0.16	88
Remote/very remote	0.01	139	0.03	16
Bottom five deciles socioeconomic disadvantage	0.44	7061	0.69	373
Share votes opposing the Voice	58.5 (14.6)	–	65.8 (14.6)	–
Share votes opposing the Voice (quartiles)				
Quartile 1: [16.2–49.3%)	0.26	4102	0.14	77
Quartile 2: [49.3–60.8%)	0.31	4990	0.21	114
Quartile 3: [60.8–72.1%)	0.24	3845	0.27	148
Quartile 4: [72.1–89.0%]	0.19	3056	0.38	205
**Outcomes**				
Self-reported health outcomes				
Poor mental health	0.14	2306	0.26	143
Fair/poor health	0.17	2654	0.22	121
Disability	0.23	3634	0.28	151
Healthcare use in past 12 months				307
Visited any healthcare provider	0.71	11,318	0.56	303
Taken any prescription medications	0.63	10,000	0.56	128
Visited a hospital	0.23	3714	0.24	346
Risk-taking behaviours				160
Smoker/ex-smoker	0.42	6740	0.64	274
Risky drinking	0.20	3162	0.29	143
Used illicit drugs	0.43	6803	0.50	121

Notes: Based on responses to the wave 21 HILDA Survey.

### Results from regression analyses

**Fig. 2:**
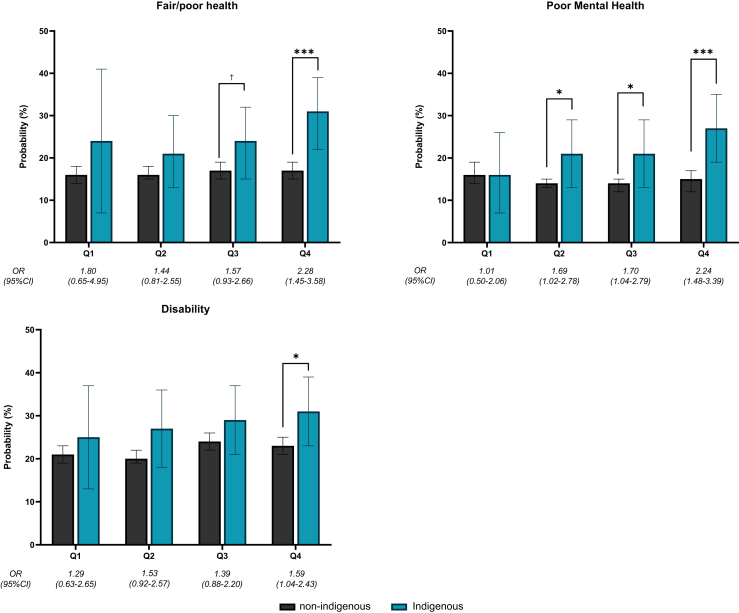
**Results from regression models for self-reported health outcomes. Notes: Probabilities estimated from marginal effects at observed values from logistic regression models. Odds ratios represent odds of Indigenous peoples reporting an outcome relative to odds for non-Indigenous Australians within same quartile of Opposition to the Voice. Within each quartile of Opposition to the Voice, pairwise significance testing performed to show whether differences between Indigenous and non-Indigenous are statistically significant: † = p < 0.10; ∗ = p < 0.05; ∗∗ = p < 0.01; ∗∗∗ = p < 0.001**.

**Fig. 3:**
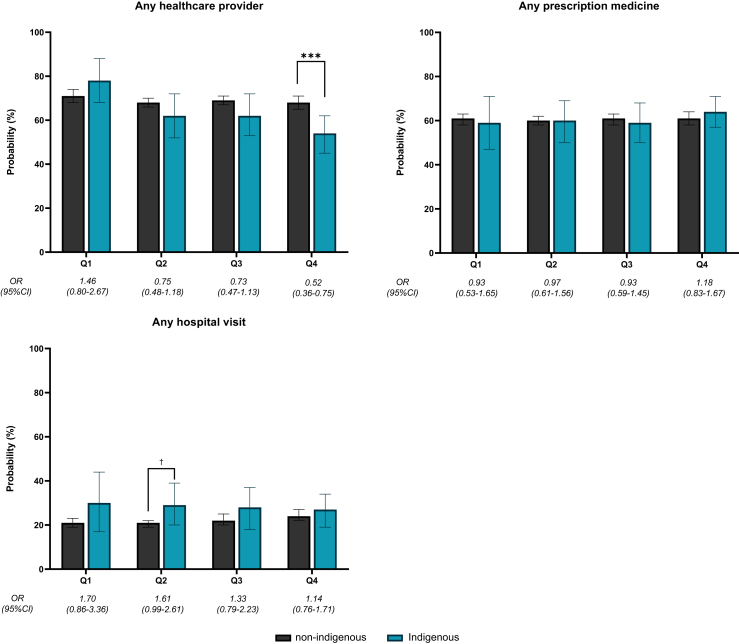
**Results from regression models for healthcare use. Notes: Probabilities estimated from marginal effects at observed values from logistic regression models. Odds ratios represent odds of Indigenous peoples reporting an outcome relative to odds for non-Indigenous Australians within same quartile of Opposition to the Voice. Within each quartile of Opposition to the Voice, pairwise significance testing performed to show whether differences between Indigenous and non-Indigenous are statistically significant: † = p < 0.10; ∗ = p < 0.05; ∗∗ = p < 0.01; ∗∗∗ = p < 0.001**.

**Fig. 4:**
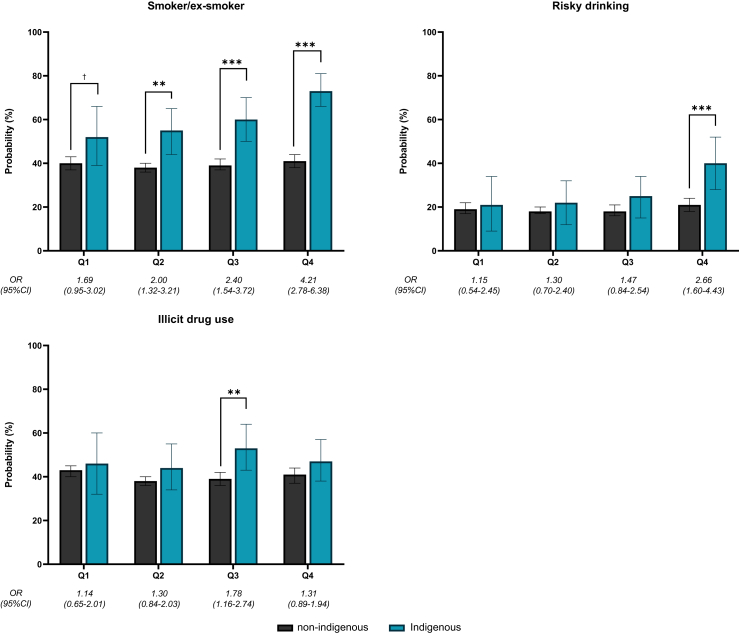
**Results from regression models for risk-taking behaviours. Notes: Probabilities estimated from marginal effects at observed values from logistic regression models. Odds ratios represent odds of Indigenous peoples reporting an outcome relative to odds for non-Indigenous Australians within same quartile of Opposition to the Voice. Within each quartile of Opposition to the Voice, pairwise significance testing performed to show whether differences between Indigenous and non-Indigenous are statistically significant: † = p < 0.10; ∗ = p < 0.05; ∗∗ = p < 0.01; ∗∗∗ = p < 0.001**.

The predicted probability of reporting health outcomes, healthcare use, and risk-taking behaviours for Indigenous and non-Indigenous Australians across different quartiles of opposition to the Voice are presented in Fig. 2 (self-reported health outcomes), Fig. 3 (healthcare use), and Fig. 4 (risk-taking behaviours), respectively.

Across all quartiles of opposition to the Voice, Indigenous peoples were generally more likely to report fair/poor health, poor mental health, and a disability compared to non-Indigenous Australians (Fig. 2). Across all health outcomes, disparities were smallest in areas where support for the Voice was highest (Q1). There is evidence of widening Indigenous health disparities in regions with higher opposition to the Voice. Compared to non-Indigenous Australians, in areas with the highest opposition to the Voice (Q4), Indigenous peoples were more likely to report fair/poor health [OR 2.28 (95% CI 1.45–3.58)], poor mental health [OR 2.24 (95% CI 1.48–3.39)], and a disability [OR 1.59 (95% CI 1.04–2.43)]. Further, the widening disparities with increasing levels of opposition to the Voice were driven by increased prevalence of poor health outcomes among Indigenous, but not non-Indigenous Australians. In areas with the lowest opposition to the Voice (Q1), the proportion of Indigenous and non-Indigenous Australians with poor mental health was similar (∼16%). In areas with the highest opposition to the Voice (Q4), the proportion of people in poor mental health was-higher for Indigenous Australians compared to non-Indigenous Australians (27% and 15%, respectively). Relative to Q1, the Q4 differences in disparities in poor mental health were statistically significant (p = 0.06).

Despite poorer self-reported health among Indigenous peoples, use of prescription medications or visiting a hospital were similar between Indigenous vs non-Indigenous people across different quartiles of opposition to the Voice (Fig. 3). The main differences were observed in visiting any healthcare provider in the last 12 months, with some evidence that higher levels of Voice opposition reduced Indigenous engagement with healthcare providers. In Q1, the proportion of Indigenous and non-Indigenous Australians who had seen any healthcare provider was similar (78% and 71% respectively) but in Q4, the proportion visiting any provider was markedly lower for Indigenous but not for non-Indigenous Australians (54% and 68%, respectively). The disparity between Indigenous and non-Indigenous Australians was statistically significant in Q4 [OR 0.52 (95% CI 0.36–0.75)]. Relative to Q1, the Q4 differences in disparities in visiting any healthcare provider were statistically significant (p < 0.01).

For risk-taking behaviours, across all levels of opposition to the Voice, Indigenous peoples were slightly more likely to report having smoked or engaged in risky drinking behaviour and illicit drug use. Disparities in risk-taking behaviours were lowest in areas with greater support for the voice (Q1) and more pronounced, for smoking and risky drinking, in regions with the highest opposition to the Voice [OR 4.21 (95% CI 2.78–6.38) for smoking, and OR 2.66 (95% CI 1.60–4.43) for risky drinking, respectively]. A dose–response relationship was apparent for smoking; higher opposition to the Voice was associated with a higher proportion of smoking for Indigenous, but not for non-Indigenous, Australians. Relative to Q1, the Q4 differences in disparities were statistically significant for smoking (p = 0.01) and risky drinking (p = 0.07).

Results were similar when including age as a continuous variable and adding additional individual- and regional-level controls; Indigenous disparities in health, healthcare, and risk-taking behaviours were lowest in areas where support for the Voice was highest (Supplementary Material SM6). In general, the explanatory power of the models also slightly increases when including these specifications (Pseduo-R2 ranging from 0.01 to 0.12 in baseline models to 0.01–0.21 in the fully adjusted models).

## Discussion

We find that Indigenous peoples living in regions of Australia with higher levels of opposition to the Voice experience poorer health outcomes, are less likely to access healthcare, and more likely to engage in risk-taking behaviours, such as smoking and binge drinking. We also find that stronger opposition to the Voice appears to have little, to no, association with these same health behaviours and outcomes for non-Indigenous Australians.

This is the first study to demonstrate how community attitudes can influence health disparities among Indigenous populations. The Voice results provide a unique opportunity to investigate regional variation in community attitudes. In particular, as votes were cast anonymously, reflect the entire voting population, and were used to directly form policy, they are less prone to biases associated with social desirability or sample selection. In turn, linking the Voice results to a representative population survey on health and health behaviours enables us to provide valuable insights on which societal-level factors predict not only health outcomes for Aboriginal and Torres Strait Islander people, but also the potential pathways through which these disparities arise.

These results are consistent with previous research linking structural discrimination, including negative community attitudes, with poorer health in racial and minority groups^2^^,^^5^, ^6^, ^7^ as well those linking interpersonal discrimination with poorer physical and mental health among Indigenous populations.^9^, ^10^, ^11^ The results also align with studies which have investigated the mechanisms through which negative social environments influence health among minority groups^3^^,^^23^, ^24^, ^25^—altogether suggesting, that, instead of engaging with healthcare, which may not be culturally safe, Indigenous peoples may be more likely to cope by engaging in risk taking behaviours such as alcohol and tobacco consumption to cope with discrimination-induced psychological stressors.

These results should be considered with the context of several limitations. First, it is important to note that the extent to which the Voice results truly reflect community-level prejudice is unclear. Some who opposed the Voice argued that the change would “not go far enough” and that stronger forms of self-determination were required.^12^ It is also possible that opposition may have been driven by a general lack of clarity or misinformation around what a ‘yes’ vote would mean. This seems plausible, considering that the political actors involved in the ‘No Campaign’ misrepresented both the intentions behind and the potential impact of the proposed constitutional reform.^12^ Despite this, there is evidence that opposition to the Voice was associated with negative attitudes towards Indigenous Australians. For example, individuals who thought that “land rights/native titles were unfair” and “if Indigenous Australians tried harder they could be just as well off as the non-Indigenous population” were more likely to vote ‘no’ in the referendum.^16^

Nevertheless, the ultimate impact of the referendum has been to undermine Indigenous self-determination in Australia. While many Australians (Indigenous and non-Indigenous) may have found the debate surrounding the Voice troubling, divisive or even exhausting, it is Aboriginal and Torres Strait Islander peoples’ health and wellbeing that is uniquely impacted following exposure to high levels of community opposition.

The representability of our results is also an important consideration, particularly as we show that Indigenous peoples within HILDA have higher levels of education and are more likely to reside in urban areas relative to Census estimates. However, given the protective health effects that factors such as income and education afford, it is likely that our results underestimate the true effect size. Further, our relatively small sample size for Aboriginal and Torres Strait Islander peoples makes it challenging to perform subgroup analyses and better pinpoint which Indigenous populations are at greatest risk of adverse health outcomes. This is crucial as within-population analysis may be equally or more important to Indigenous communities. This also reinforces the need for strong Data Sovereignty and Governance processes to help ensure that research insights are relevant to Aboriginal and Torres Strait Islander peoples. For example, it remains unclear how contributions from Aboriginal and Torres Strait Islander peoples and governance bodies are embedded into the development and ongoing data collection of HILDA.

As our preferred models deliberately exclude various individual-level factors which may be impacted by discrimination (and affect health) it is important to acknowledge that our models explain only small amounts of variation in the dependent variables. There are also likely numerous unobserved factors that predict health outcomes which are not able to be readily tested in our sample. Inadequate representation of Indigenous peoples within population level datasets remains an ongoing challenge, particularly for studying health equity.^26^^,^^27^

Moreover, acknowledging its prior application to Indigenous health research,^28^ it is important to recognise that HILDA was not designed by, or for, Indigenous Australians. Consequently, HILDA does not capture dimensions of health important for Indigenous Australians.^29^ This again underscores the need for self-determination processes to be embedded in health data.

Nevertheless, we believe it is possible to work within a critical allyship framework^30^ to unlock culturally relevant insights guided by Indigenous scholars with expertise in working with mainstream health datasets.

It is also possible that the robustness of our findings could be affected by reporting error in self-reported health measures, particularly if there were systematic differences in reporting between Indigenous and non-Indigenous participants, which could lead to information bias. For example, measuring the prevalence of certain health conditions may not be appropriate for populations who are less likely to engage with mainstream healthcare. Improved identification of Indigenous peoples in larger population health datasets, and validation of health measures, will therefore be essential for ongoing monitoring, advocacy, and reform evaluation. Consultation with Indigenous community is also essential to ensure questions, self-reported or otherwise, adequately capture health and wellbeing.

Finally, it is important to note that this analysis is cross-sectional and based on individuals' ‘exposure’ to a proxy for community attitudes, as measured in 2023, mapped to health measures reported in 2021. This approach assumes that these underlying attitudes were relatively constant and thus does not adequately account for selective migration,^3^ the impact of the referendum itself, nor other changes in societal-level conditions which may disadvantage or constrain the health and wellbeing of Indigenous peoples. Future research should therefore consider other measures of societal-level conditions that proxy structural discrimination at distinct time points and investigate how changes in these conditions, including the discourse around the referendum in itself, may influence health, and other social outcomes, among Indigenous Australians.

Despite these limitations, our study is the first to provide empirical population-level evidence that Indigenous health disparities are larger in areas with greater opposition to the Voice. These results have clear and pressing implications for social and health policy. First, our results suggest that in areas with greater opposition to the Voice, Indigenous peoples are not accessing healthcare at levels commensurate to need. It is possible that, in these regions, there is a scope for more culturally diverse healthcare or campaigns around when one should seek care, especially for illnesses associated with increased stress. However, an important shift in designing these pathways is the need for greater attention to how community-level attitudes shape individual health behaviors, risk taking and otherwise. Eliminating structural discrimination requires an understanding of the multiple levels of influence (societal, community, interpersonal, individual) which drive Indigenous health disparities in Australia. Strengthening anti-discrimination laws and community engagement and dialogue are likely to play an important role in reducing discrimination against Indigenous peoples, however, in light of the failed referendum, multi-level health policy and programs are urgently needed. Other structural and institutional reforms, such as treaties or other constructive arrangements, are required to reduce persistent and unfair health disparities experienced by Indigenous populations.

## Contributors

K Saxby; Conceptualization, Data curation, Formal analysis, Funding acquisition, Investigation, Methodology, Project administration, Writing—original draft, Writing—review and editing.

Z Aitken; Conceptualization, Methodology, Data curation, Formal analysis, Investigation, Writing—review and editing.

L Burchill; Conceptualization, Methodology, Writing—review and editing.

Y Zhang; Funding acquisition, Conceptualization, Methodology, Writing—review and editing.

## Data sharing statement

Data is available upon request to the Department of Social Services.

## Editor note

The Lancet Group takes a neutral position with respect to territorial claims in published maps and institutional affiliations.

## Declaration of interests

There are no relevant interests to declare.
