# Supplementary material for: Community attitudes and Indigenous health disparities: evidence from Australia's Voice referendum
Source: Lancet Reg Health West Pac. 2024 Aug 1;50:101154. doi: 10.1016/j.lanwpc.2024.101154 (PMC11472229; doi:10.1016/j.lanwpc.2024.101154)
Supplement: Supplementary Material [file mmc1.docx]

# Supplementary Material

### Engagement with Aboriginal and Torres Strait Islander peoples and organisations

Engagement with Aboriginal and Torres Strait Islander peoples and communities central to this research and was embedded throughout all stages of research design, analysis, and interpretation. In particular, the insights from our collaboration prompted: key changes in selection of outcome measures, refinement to the language to take a strengths-based perspective, and ensuring that language was centred around Indigenous experiences. This research has employed multifaceted engagement has been documented, in part, through the Aboriginal and Torres Strait Islander Quality Assessment Tool developed by Harfield et al. (2020). This information is provided below.

|  | **Yes** | **Partially** | **No** | **Unclear** |
| --- | --- | --- | --- | --- |
| **1. Did the research respond to a need or priority determined by the community?**  This responds to community and sector needs through several different facets.  First, this research aligns with sector-based priorities as outlined in **NACCHO Strategic Directions 2023–2025**. Specifically, this aligns with Strategy 1 to “strengthen NACCHO’s national influence to increase investment in Aboriginal and Torres Strait Islander comprehensive primary health care,” and Strategy 4 “to further strengthen NACCHO’s knowledge-base and capability of the sector” as it provides place-based evidence as to where greater investment is needed to close inequalities in health and healthcare. The latter was also explicitly called for in a recent report sent to government in which the research team was engaged by NACCHO to provide specific evidence on healthcare use and health outcomes among Indigenous populations.  Specific aspects also respond to priorities within the **National Aboriginal and Torres Strait Islander Health Plan 2021–2031** (Priority 8 “Identify and eliminate racism,” “ensure that care is place-based,” and “ensure mainstream services address racism and provide culturally safe and responsive care”) and the **National Agreement** approaches to identify “social determinants of health” and to “support Aboriginal and Torres Strait Islander communities to analyse and use regional specific data to help drive their own development and discussions with governments” (Partnership Priority Reform 3 “Shared Access to Data and Information at a Regional Level”). This work also aligns with priority reforms to “transform government organisations” as the research outputs from this work will be used to directly inform the provision of services and healthcare, including at the federal level, by providing direct information on place-based health inequalities.  This research was also conducted in response to the emerging issue highlighted by the Voice Referendum itself, whereby members of the community reported experiencing higher levels of interpersonal discrimination. This was explicitly noted by Indigenous leaders (Ian Anderson, Yin Paradies, Marcia Langton, Ray Lovett, Tom Calma) in their article titled “Racism and the 2023 Australian constitutional referendum.” | X |  |  |  |
| **2. Was community consultation and engagement appropriately inclusive?**  Study author KS has longstanding collaborations with NACCHO (since 2017). KS research with NACCHO has largely focused on using administrative data to show inequalities in primary healthcare use. This research involved collaboration with NACCHO members, and in particular, their Clinical Leadership team during 2021/22. As a result of this engagement, KS has continued to use administrative data to provide evidence on area-level health and healthcare inequalities experienced by Indigenous peoples. This research was used for NACCHO’s 2023 reporting to government. In this reporting, NACCHO called for information on where healthcare access by Indigenous populations was inequitably lower relative to non-Indigenous Australians. This led the research team to consider survey-based measures of healthcare use and health and wellbeing. In combination with the referendum, this led to a broader consideration of societal-level factors.  We then sought to complement NACCHO priorities with the lived experience critical for this research. To this end, we discussed initial ideas with A/Prof Luke Burchill – a Yorta Yorta man with expertise in Indigenous health and ethical conduct in research with First Nations peoples. Discussions continued throughout the project, and Luke Burchill joined the study team as a co-author. The insights from our collaboration prompted key changes in the manuscript including refinements to the framing, language, and discussion to ensure that language was centred around Indigenous experiences and that outcomes and analyses were appropriate. Specific changes which were implemented also included expanding the analyses to consider a strength-based approach when considering risk-taking behaviours (and framing these as an indicators of reduced self-care and potential coping strategies), and portraying how opposition to the Voice varied for outcomes among non-Indigenous Australians.  It is important to note that consultation and engagement is ongoing. This research is part of an ongoing collaborations with NACCHO to document inequitable healthcare access at the regional level and to explore how access to culturally appropriate services reduces health inequalities among Indigenous communities. Alongside Indigenous leadership in the research and alignment with NACCHO and sector priorities, KS is undertaking a six-week secondment to an ACCHO via the Jawun secondment program in 2024. | X |  |  |  |
| **3. Did the research have Aboriginal and Torres Strait Islander research leadership?** A Yorta Yorta man Luke Burchill was involved in all stages of research conceptualisation, design, and analysis and has oversight on the integrity of the research and its output. This research is also a part of a larger body of work we are seeking to conduct which includes A/Prof Luke Burchill as a CI and Mike Stephens of NACCHO as a CI. | X |  |  |  |
| **4. Did the research have Aboriginal and Torres Strait Islander governance?**  A/Prof Luke Burchill ensured that community protocols were followed and enable relevant cultural and contextual knowledge to inform the research. Consultation and engagement is ongoing. Alongside Indigenous leadership in the research and alignment with NACCHO and sector priorities, KS is undertaking a secondment to an ACCHO via the Jawun secondment program. |  | X |  |  |
| **5. Were local community protocols respected and followed?** It is our understanding that community protocols are always respected during collection of the HILDA data (personal communication, Roger Wilkins, 2024). | X |  |  |  |
| **6. Did the researchers negotiate agreements in regards to rights of access to Aboriginal and Torres Strait Islander peoples’ existing intellectual and cultural property?**  The data used in this study is deidentified and sourced from the Household, Income and Labour Dynamics in Australia (HILDA) Survey. The HILDA data is funded by the Department of Social Services (DSS) and administered by the Melbourne Institute (MI). Based on team correspondence with the data custodians, it is our understanding that DSS alignment with Data Sovereignty is achieved via ongoing guidance from Aboriginal and Torres Strait Islander stakeholders, alignment with Australian Institute of Aboriginal and Torres Strait Islander Studies Code of Ethics for Aboriginal and Torres Strait Islander Research, and that culturally safe practices are employed around collection, governance, and release of data about Aboriginal and Torres Strait Islander peoples. The DSS does not provide this information in a consolidated format however so how specific procedures are followed is unclear. |  |  |  | X |
| **7. Did the researchers negotiate agreements to protect Aboriginal and Torres Strait Islander peoples' ownership of intellectual and cultural property created through the research?**  This research would not be possible without the contribution of the Aboriginal and Torres Strait Islander peoples in HILDA. In accordance with the Ethical conduct in research with Aboriginal and Torres Strait Islander Peoples and communities: Guidelines for researchers and stakeholders document, through data collection in HILDA, all participant decisions to participate are voluntary, participants are fully informed, and participants understand the information. At the University of Melbourne, all published material must abide by the National Health and Medical Research Council’s Values and Ethics: Guidelines for Ethical Conduct in Aboriginal and Torres Strait Islander Health Research. While this research article adheres to these guidelines (as evidenced by the ethics approval and explicit alignment with these guidelines), there remains limited oversight on how contributions of the Aboriginal and Torres Strait Islander peoples, researchers, research participants and governance bodies are embedded in the development and ongoing collection of HILDA. There is also no information provided about control over intellectual and cultural property, adherence to cultural protocols, or use of Indigenous methodologies. |  | X |  |  |
| **8. Did Aboriginal and Torres Strait Islander peoples and communities have control over the collection and management of research materials?**  The data used in this study is deidentified and sourced from the Household, Income and Labour Dynamics in Australia (HILDA) Survey. The HILDA data is funded by the Department of Social Services (DSS) and administered by the Melbourne Institute (MI). It is our understanding that DSS alignment with Data Sovereignty is achieved via ongoing guidance from Aboriginal and Torres Strait Islander stakeholders and that culturally safe practices are employed around collection, governance, and release of data about Aboriginal and Torres Strait Islander peoples. It is noted in the assessment tool that development of the survey protocol and administration should be done “in partnership with Aboriginal and Torres Strait Islander peoples for the respectful and appropriate collection and management of all biological and non-biological research materials.” However, it is not clear how this process is carried out with respect to the HILDA Survey. This component of the tool also requires that “consent processes give the participants control over how their data and samples will be managed.” The latter is achieved via informed consent processes: all participant decisions to participate are voluntary, participants are fully informed, and participants understand the information and how the HILDA responses will be used before consenting. |  | X |  |  |
| **9. Was the research guided by an Indigenous research paradigm?**  The research methodology and methods must reflect the community values, priorities and perspectives of research participants and their communities. In particular, this research acknowledges that health and wellbeing are complex and interconnected and require multiple research methods, and refinement of the research, to present health inequalities at a SA3 level. This study was guided by an Indigenous research approach. One of the Chief Investigators A/Prof Burchill (Yorta Yorta) ensured that we placed value on Indigenous knowledge and privileged Indigenous voices and Indigenous experiences throughout the research process. | X |  |  |  |
| **10. Does the research take a strengths-based approach, acknowledging and moving beyond practices that have harmed Aboriginal and Torres Strait peoples in the past?**  A strength-based approach was employed, in particular, pertaining to the research question on mental health and risk-taking behaviour. This framing was specifically altered to avoid a deficit approach and convey that these behaviours may have been induced by experiences of interpersonal discrimination and as reduced self-care rather than framed as ‘risk factors’. These measures and the framing was entirely informed by A/Prof Burchill. This language was further refined based on important commentary provided by the Lancet Regional Health Western Pacific editorial review team. | X |  |  |  |
| **11. Did the researchers plan and translate the findings into sustainable changes in policy and/or practice?**  The research solutions proposed in the manuscript are informed by A/Prof Burchill. Implementation and translation of this research is a part of ongoing, multi-year, engagement processes. Initial phases of engagement with NACCHO have centred around using administrative data to show that healthcare access is lower among Indigenous populations relative to non-Indigenous Australians. That research has since been published and used by NACCHO in advocacy in government reporting. The present research supplements this by providing area level evidence on where healthcare use is inequitably low. Processes of translation and engagement are ongoing however this research has been submitted as evidence as a part of the Yoorook Justice Commission. Funding has been sought for the establishment of an Advisory Committee (AC) with representatives from the community, for-purpose organisations, and government. A Jawun secondment is scheduled which will focus on area-level priorities. Final outcomes are expected by September 2024. | X |  |  |  |
| **12. Did the research benefit the participants and Aboriginal and Torres Strait Islander communities?**  This research supplements a large body of work being carried out in consultation with NACCHO which will be used for ongoing advocacy. Implementation and translation of this research is a part of ongoing, multi-year, engagement processes. |  | X |  |  |
| **13. Did the research demonstrate capacity strengthening for Aboriginal and Torres Strait Islander individuals?**  This research is a part of broader engagement and mutual capacity building with NACCHO (and an ACCHO to be identified later this year). For several years, KS has provided pro bono consulting services to NACCHO with a focus on embedding empirical and health economics lens into NACCHO reporting to government. This evidence is crucial for demonstrating areas where healthcare access is inequitably low. As a part of this broader engagement, this project is aiming to obtain funding for an Aboriginal and Torres Strait Islander student (Masters or PhD student). |  | X |  |  |
| **14. Did everyone involved in the research have opportunities to learn from each other?**  Through this research and broader research commitments with NACCHO, the non-Indigenous research team grew in their capacity to conduct research with Aboriginal researchers. This was achieved through group meetings, shared report development, and the shared development and oversight of this particular manuscript. | X |  |  |  |

### HILDA vs Census sample

|  | **Indigenous  (HILDA 2021)** | **Indigenous  (Census 2021)** |
| --- | --- | --- |
|  | **(n=544)** | **(n=461,745)** |
|  | Mean/Prop. | Mean/Prop. |
| ***Individual characteristics*** |  |  |
| Age | 35.9 | 38.2 |
| Male | 0.44 | 0.47 |
| *Educational attainment* |  |  |
| Less than High school | 0.38 | 0.26 |
| High school or equivalent^A^ | 0.45 | 0.45 |
| Bachelor or above | 0.16 | 0.08 |
| ***Regional characteristics*** |  |  |
| Major cities | 0.47 | 0.44 |
| Inner regional | 0.35 | 0.25 |
| Outer regional | 0.16 | 0.19 |
| Remote / very remote | 0.03 | 0.12 |
| Bottom five deciles socioeconomic deprivation | 0.69 | 0.72 |
| Share votes opposing the Voice | 65.8 | 67.1 |
| *Share votes opposing the Voice (quartiles)* |  |  |
| Quartile 1 [16.2-49.3%) | 0.14 | 0.08 |
| Quartile 2 [49.3-60.8%) | 0.21 | 0.18 |
| Quartile 3 [60.8-72.1%) | 0.27 | 0.37 |
| Quartile 4 [72.1-89.0%] | 0.38 | 0.36 |

Notes : Census sample restricted to those aged 15 and above at the time of the Census. Sourced from PLIDA data (ABS, 2022) with sample construction conducted as per (Saxby et al., 2023). A) equivalent refers to those who completed professional qualification, certificate, or Technical and Further Education (TAFE).

### Logistic regression model

To test whether Indigenous disparities in health outcomes vary across different levels of opposition to the Voice, we apply a logistic regression model of the following form:

$$\ln\left( \frac{p_{y_{ir}}}{1-p_{y_{ir}}} \right)= \beta_{0}+ \sum_{k=2}^{4} \beta_{k}\left( \boldsymbol{V}_{\boldsymbol{r}} \right)+\sum_{l=1}^{4} \beta_{l}\left( I_{i}\times\boldsymbol{V}_{\boldsymbol{r}} \right)+\boldsymbol{\gamma}_{r}+\boldsymbol{X}_{i}$$

Where $p_{y_{ir}}$ represents the probability that individual $i$ living in SA3-region $r$ reports the outcome of interest $y$(e.g., poor mental health, whether visited healthcare provider). $I_{i}$ is a binary indicator equal to one if the individual is Indigenous; $\boldsymbol{X}_{\boldsymbol{i}}$ is a vector of individual level controls; $\boldsymbol{V}_{r}$ is a categorical variable representing the quartile of percentage of opposition to the Voice in region $r$, and $\boldsymbol{\gamma}_{r}$ represents a vector of regional-level confounders.

The coefficients in $\beta_{k}$ describe how the outcome changes for non-Indigenous Australians across different quartiles of opposition to the Voice. The coefficients in $\beta_{l}$ present the cross-level interaction between Indigenous status and the quartile of opposition to the Voice. Thus, $\beta_{l}$ describes how Indigenous disparities in outcomes varies across different levels of opposition to the Voice, relative to non-Indigenous Australians.

To adjust for potential regional-level confounders, in $\boldsymbol{\gamma}_{r}$ we control for area-level disadvantage, population density, and state/territory fixed effects.

In $\boldsymbol{X}_{i}$, we also control for age categories (‘15-39 years,’ ‘40-59 years,’ ‘60 plus years’) and sex (‘male’, ‘female’).

### Full results logistic regression models

Table SM4.1 – Logistic regression results for health outcomes

|  | **Fair/poor health** | | **Poor mental health** | | **Disability** | |
| --- | --- | --- | --- | --- | --- | --- |
|  | $\boldsymbol{\beta}$ | **95%CI** | $\boldsymbol{\beta}$ | **95%CI** | $\boldsymbol{\beta}$ | **95%CI** |
| Age group |  |  |  |  |  |  |
| 15-39 years (ref) | 1.00 | 1.00 | 1.00 | 1.00 | 1.00 | 1.00 |
| 40-59 years | 1.85 | (1.57-2.18) | 0.70 | (0.61-0.80) | 1.92 | (1.67-2.20) |
| >=60 years | 4.30 | (3.65-5.05) | 0.46 | (0.39-0.54) | 5.48 | (4.84-6.20) |
| Female (ref) | 1.00 | (1.00-1.00) | 1.00 | (1.00-1.00) | 1.00 | (1.00-1.00) |
| Male | 0.90 | (0.79-1.01) | 0.68 | (0.60-0.77) | 0.89 | (0.80-0.98) |
| NSW (ref) | 1.00 | (1.00-1.00) | 1.00 | (1.00-1.00) | 1.00 | (1.00-1.00) |
| VIC | 0.99 | (0.83-1.17) | 1.17 | (0.99-1.39) | 1.15 | (1.00-1.33) |
| QLD | 1.18 | (0.98-1.43) | 0.99 | (0.81-1.20) | 1.02 | (0.86-1.20) |
| SA | 1.21 | (0.98-1.49) | 1.10 | (0.87-1.40) | 1.34 | (1.11-1.61) |
| WA | 1.09 | (0.88-1.36) | 1.00 | (0.80-1.25) | 1.48 | (1.23-1.79) |
| TAS | 1.05 | (0.78-1.40) | 1.07 | (0.78-1.48) | 1.59 | (1.25-2.03) |
| NT | 1.77 | (0.83-3.77) | 0.76 | (0.23-2.50) | 1.21 | (0.60-2.44) |
| ACT | 0.92 | (0.57-1.48) | 0.99 | (0.63-1.56) | 1.54 | (1.05-2.25) |
| Socioeconomic deprivation quintile=1 (ref) | 1.00 | (1.00-1.00) | 1.00 | (1.00-1.00) | 1.00 | (1.00-1.00) |
| Socioeconomic deprivation quintile=2 | 0.89 | (0.72-1.10) | 1.06 | (0.85-1.31) | 0.88 | (0.74-1.05) |
| Socioeconomic deprivation quintile=3 | 0.83 | (0.68-1.02) | 1.00 | (0.81-1.25) | 0.72 | (0.60-0.85) |
| Socioeconomic deprivation quintile=4 | 0.68 | (0.54-0.86) | 0.84 | (0.64-1.09) | 0.56 | (0.46-0.68) |
| Socioeconomic deprivation quintile=5 | 0.46 | (0.35-0.62) | 0.72 | (0.52-0.98) | 0.40 | (0.31-0.52) |
| Population density | 1.00 | (1.00-1.00) | 1.00 | (1.00-1.00) | 1.00 | (1.00-1.00) |
| Q1 opposition (ref) | 1.00 | 1.00 | 1.00 | 1.00 | 1.00 | 1.00 |
| Q2 opposition | 1.03 | (0.84-1.26) | 0.84 | (0.68-1.03) | 0.95 | (0.80-1.13) |
| Q3 opposition | 1.09 | (0.85-1.40) | 0.80 | (0.61-1.05) | 1.18 | (0.95-1.46) |
| Q4 opposition | 1.10 | (0.83-1.47) | 0.88 | (0.65-1.18) | 1.12 | (0.89-1.42) |
| Q1 opposition x Indigenous | 1.80 | (0.65-4.95) | 1.01 | (0.50-2.06) | 1.29 | (0.63-2.65) |
| Q2 opposition x Indigenous | 1.44 | (0.81-2.55) | 1.69 | (1.02-2.78) | 1.53 | (0.92-2.57) |
| Q3 opposition x Indigenous | 1.57 | (0.93-2.66) | 1.70 | (1.04-2.79) | 1.39 | (0.88-2.20) |
| Q4 opposition x Indigenous | 2.28 | (1.45-3.58) | 2.24 | (1.48-3.39) | 1.59 | (1.04-2.43) |
| *N* | *14,619* |  | *14,704* |  | *15,870* |  |
| *Pseudo-R2* | *0.07* |  | *0.02* |  | *0.10* |  |

Notes: Exponentiated coefficients.

Table SM4.2 – Logistic regression results for healthcare use in past 12 months

|  | **Visited any health provider** | | **Taken any prescription medications** | | **Visited hospital** | |
| --- | --- | --- | --- | --- | --- | --- |
|  | $\boldsymbol{\beta}$ | **95%CI** | $\boldsymbol{\beta}$ | **95%CI** | $\boldsymbol{\beta}$ | **95%CI** |
| Age group |  |  |  |  |  |  |
| 15-39 years (ref) | 1.00 | 1.00 | 1.00 | 1.00 | 1.00 | 1.00 |
| 40-59 years | 1.54 | (1.38-1.72) | 2.01 | (1.80-2.23) | 1.18 | (1.03-1.35) |
| >=60 years | 3.17 | (2.79-3.61) | 8.00 | (6.97-9.18) | 2.24 | (1.98-2.52) |
| Female (ref) |  |  |  |  |  |  |
| Male | 0.57 | (0.51-0.63) | 0.56 | (0.51-0.62) | 0.72 | (0.65-0.79) |
| NSW (ref) | 1.00 | (1.00-1.00) | 1.00 | (1.00-1.00) | 1.00 | (1.00-1.00) |
| VIC | 1.13 | (0.98-1.29) | 1.18 | (1.03-1.35) | 1.11 | (0.97-1.26) |
| QLD | 1.30 | (1.13-1.50) | 1.33 | (1.15-1.54) | 1.19 | (1.01-1.40) |
| SA | 1.31 | (1.08-1.58) | 1.31 | (1.09-1.58) | 1.09 | (0.91-1.30) |
| WA | 1.15 | (0.96-1.39) | 1.25 | (1.04-1.49) | 1.15 | (0.96-1.37) |
| TAS | 1.10 | (0.87-1.39) | 1.25 | (0.98-1.58) | 1.34 | (1.04-1.72) |
| NT | 1.78 | (1.06-2.98) | 0.72 | (0.40-1.30) | 0.75 | (0.37-1.54) |
| ACT | 1.76 | (1.20-2.58) | 1.32 | (0.94-1.85) | 1.05 | (0.71-1.54) |
| Socioeconomic deprivation quintile=1 (ref) | 1.00 | (1.00-1.00) | 1.00 | (1.00-1.00) | 1.00 | (1.00-1.00) |
| Socioeconomic deprivation quintile=2 | 1.12 | (0.94-1.33) | 1.14 | (0.96-1.37) | 1.05 | (0.87-1.26) |
| Socioeconomic deprivation quintile=3 | 0.95 | (0.80-1.13) | 0.96 | (0.81-1.15) | 1.12 | (0.94-1.34) |
| Socioeconomic deprivation quintile=4 | 1.12 | (0.92-1.37) | 1.01 | (0.83-1.23) | 1.24 | (1.02-1.51) |
| Socioeconomic deprivation quintile=5 | 1.33 | (1.05-1.70) | 0.90 | (0.71-1.14) | 1.16 | (0.92-1.46) |
| Population density | 1.00 | (1.00-1.00) | 1.00 | (1.00-1.00) | 1.00 | (1.00-1.00) |
| Q1 opposition (ref) | 1.00 | (1.00-1.00) | 1.00 | (1.00-1.00) | 1.00 | (1.00-1.00) |
| Q2 opposition | 0.87 | (0.74-1.02) | 0.98 | (0.84-1.14) | 0.99 | (0.85-1.16) |
| Q3 opposition | 0.91 | (0.74-1.11) | 1.00 | (0.82-1.22) | 1.11 | (0.90-1.37) |
| Q4 opposition | 0.88 | (0.69-1.11) | 1.01 | (0.81-1.27) | 1.23 | (0.97-1.56) |
| Q1 opposition x Indigenous | 1.46 | (0.80-2.67) | 0.93 | (0.53-1.65) | 1.70 | (0.86-3.36) |
| Q2 opposition x Indigenous | 0.75 | (0.48-1.18) | 0.97 | (0.61-1.56) | 1.61 | (0.99-2.61) |
| Q3 opposition x Indigenous | 0.73 | (0.47-1.13) | 0.93 | (0.59-1.45) | 1.33 | (0.79-2.23) |
| Q4 opposition x Indigenous | 0.52 | (0.36-0.75) | 1.18 | (0.83-1.67) | 1.14 | (0.76-1.71) |
| *N* | *15,917* |  | *15,920* |  | *15,921* |  |
| *Pseudo-R2* | *0.05* |  | *0.12* |  | *0.03* |  |

Notes: Exponentiated coefficients.

Table SM4.3 – Logistic regression results for risk-taking behaviours

|  | **Smoker/ex-smoker** | | **Risky drinking** | | **Any illicit drug use** | |
| --- | --- | --- | --- | --- | --- | --- |
|  | $\boldsymbol{\beta}$ | **95%CI** | $\boldsymbol{\beta}$ | **95%CI** | $\boldsymbol{\beta}$ | **95%CI** |
| Age group |  |  |  |  |  |  |
| 15-39 years (ref) | 1.00 | (1.00-1.00) | 1.00 | (1.00-1.00) | 1.00 | (1.00-1.00) |
| 40-59 years | 1.96 | (1.74-2.19) | 0.96 | (0.84-1.11) | 1.04 | (0.93-1.16) |
| >=60 years | 2.15 | (1.93-2.41) | 0.48 | (0.41-0.56) | 1.10 | (0.99-1.22) |
| Female (ref) |  |  |  |  |  |  |
| Male | 1.55 | (1.41-1.69) | 1.87 | (1.66-2.12) | 0.99 | (0.90-1.08) |
| NSW (ref) | 1.00 | (1.00-1.00) | 1.00 | (1.00-1.00) | 1.00 | (1.00-1.00) |
| VIC | 1.04 | (0.91-1.18) | 1.06 | (0.89-1.27) | 1.16 | (1.03-1.31) |
| QLD | 1.00 | (0.87-1.15) | 1.16 | (0.97-1.38) | 1.25 | (1.09-1.45) |
| SA | 0.91 | (0.78-1.07) | 1.10 | (0.87-1.38) | 1.23 | (1.05-1.45) |
| WA | 1.01 | (0.86-1.20) | 1.28 | (1.02-1.60) | 1.41 | (1.20-1.65) |
| TAS | 1.57 | (1.25-1.97) | 1.35 | (0.99-1.83) | 1.36 | (1.09-1.69) |
| NT | 1.72 | (1.02-2.91) | 2.28 | (1.24-4.20) | 1.37 | (0.77-2.43) |
| ACT | 0.91 | (0.63-1.31) | 0.61 | (0.38-0.97) | 0.94 | (0.68-1.31) |
| Socioeconomic deprivation quintile=1 (ref) | 1.00 | (1.00-1.00) | 1.00 | (1.00-1.00) | 1.00 | (1.00-1.00) |
| Socioeconomic deprivation quintile=2 | 1.02 | (0.87-1.20) | 1.12 | (0.91-1.38) | 0.94 | (0.79-1.10) |
| Socioeconomic deprivation quintile=3 | 0.90 | (0.77-1.05) | 1.10 | (0.89-1.37) | 0.80 | (0.68-0.93) |
| Socioeconomic deprivation quintile=4 | 1.00 | (0.84-1.19) | 1.20 | (0.95-1.52) | 0.89 | (0.75-1.07) |
| Socioeconomic deprivation quintile=5 | 0.74 | (0.59-0.91) | 1.01 | (0.75-1.34) | 0.83 | (0.67-1.02) |
| Population density | 1.00 | (1.00-1.00) | 1.00 | (1.00-1.00) | 1.00 | (1.00-1.00) |
| Q1 opposition (ref) | 1.00 | (1.00-1.00) | 1.00 | (1.00-1.00) | 1.00 | (1.00-1.00) |
| Q2 opposition | 0.93 | (0.80-1.07) | 0.93 | (0.77-1.12) | 0.83 | (0.72-0.95) |
| Q3 opposition | 0.97 | (0.81-1.17) | 0.94 | (0.73-1.22) | 0.86 | (0.72-1.04) |
| Q4 opposition | 1.04 | (0.84-1.29) | 1.11 | (0.82-1.49) | 0.92 | (0.75-1.13) |
| Q1 opposition x Indigenous | 1.69 | (0.95-3.02) | 1.15 | (0.54-2.45) | 1.14 | (0.65-2.01) |
| Q2 opposition x Indigenous | 2.00 | (1.28-3.10) | 1.30 | (0.70-2.40) | 1.30 | (0.84-2.03) |
| Q3 opposition x Indigenous | 2.40 | (1.54-3.72) | 1.46 | (0.84-2.54) | 1.78 | (1.16-2.74) |
| Q4 opposition x Indigenous | 4.21 | (2.78-6.38) | 2.66 | (1.60-4.43) | 1.31 | (0.89-1.94) |
| *N* | *14,658* |  | *11,578* |  | *14,585* |  |
| *Pseudo-R2* | *0.04* |  | *0.03* |  | *0.01* |  |

Notes: Exponentiated coefficients.

### Full results predicted probabilities

Please note, in the below tables, we rescaled all predicted probabilities from 0-1 to percentages (0-100).

Table SM5.1 – Probability non-Indigenous and Indigenous Australians with fair/poor health, poor mental health, and disability across different quartiles of opposition to the Voice

| **Outcomes** | **Probability** | | **OR** | **p-value for test of equivalence to Q1** |
| --- | --- | --- | --- | --- |
|  | **Non-Indigenous** | **Indigenous** |  |  |
| ***Fair/poor health*** |  |  |  |  |
| Quartile 1 | 16 (14-18) | 24 (7-41) | 1.80 (0.65-4.95) | - |
| Quartile 2 | 16 (15-18) | 21 (13-30) | 1.44 (0.81-2.55) | p=0.71 |
| Quartile 3 | 17 (15-19) | 24 (15-32) | 1.57 (0.93-2.66) | p=0.82 |
| Quartile 4 | 17 (15-19) | 31 (22-39) | 2.28 (1.45-3.58) | p=0.67 |
| ***Poor mental health*** |  |  |  |  |
| Quartile 1 | 16 (14-19) | 16 (7-26) | 1.01 (0.50-2.06) | - |
| Quartile 2 | 14 (13-15) | 21 (13-29) | 1.69 (1.02-2.78) | p=0.24 |
| Quartile 3 | 14 (12-15) | 21 (13-29) | 1.70 (1.04-2.79) | p=0.24 |
| Quartile 4 | 15 (12-17) | 27 (19-35) | 2.24 (1.48-3.39) | p=0.06^†^ |
| ***Disability*** |  |  |  |  |
| Quartile 1 | 21 (19-23) | 25 (13-37) | 1.29 (0.63-2.65) | - |
| Quartile 2 | 20 (19-22) | 27 (18-36) | 1.53 (0.92-2.57) | p=0.70 |
| Quartile 3 | 24 (22-26) | 29 (21-37) | 1.39 (0.88-2.20) | p=0.86 |
| Quartile 4 | 23 (21-25) | 31 (23-39) | 1.59 (1.04-2.43) | p=0.62 |

Notes: Estimated prevalence estimated from marginal effects of multilevel logistic regression models. 95% confidence intervals presented in brackets. Hypothesis testing present p-values for test of equivalence to Q1: † = p < 0.10; *=p < 0.05; ** = p < 0.01; *** = p < 0.001.

Table SM5.2 – Probability non-Indigenous and Indigenous Australians using healthcare across different quartiles of opposition to the Voice

| **Outcomes** | **Probability** | | **OR** | **p-value for test of equivalence to Q1** |
| --- | --- | --- | --- | --- |
|  | **Non-Indigenous** | **Indigenous** |  |  |
| ***Any healthcare provider*** |  |  |  |  |
| Quartile 1 | 71 (68-74) | 78 (68-88) | 1.46 (0.80-2.67) | - |
| Quartile 2 | 68 (66-70) | 62 (52-72) | 0.75 (0.48-1.18) | p=0.08^†^ |
| Quartile 3 | 69 (67-71) | 62 (53-72) | 0.73 (0.47-1.13) | p=0.07^†^ |
| Quartile 4 | 68 (65-71) | 54 (45-62) | 0.52 (0.36-0.75) | p=0.004** |
| ***Any prescription medicine*** |  |  |  |  |
| Quartile 1 | 61 (58-63) | 59 (47-71) | 0.93 (0.53-1.65) | - |
| Quartile 2 | 60 (58-62) | 60 (50-69) | 0.97 (0.61-1.56) | p=0.90 |
| Quartile 3 | 61 (58-63) | 59 (50-68) | 0.93 (0.59-1.45) | p=0.47 |
| Quartile 4 | 61 (58-64) | 64 (57-71) | 1.18 (0.83-1.67) | p=0.49 |
| ***Any hospital visit*** |  |  |  |  |
| Quartile 1 | 21 (19-23) | 30 (17-44) | 1.70 (0.86-3.36) | - |
| Quartile 2 | 21 (19-22) | 29 (20-39) | 1.61 (0.99-2.61) | p=0.88 |
| Quartile 3 | 22 (20-25) | 28 (18-37) | 1.33 (0.79-2.23) | p=0.57 |
| Quartile 4 | 24 (22-27) | 27 (19-34) | 1.14 (0.76-1.71) | p=0.32 |

Notes: Estimated prevalence estimated from marginal effects of multilevel logistic regression models. 95% confidence intervals presented in brackets. Hypothesis testing present p-values for test of equivalence to Q1: † = p < 0.10; *=p < 0.05; ** = p < 0.01; *** = p < 0.001.

Table SM5.3 – Probability and Odd Ratios of risk-taking behaviour across different quartiles of opposition to the Voice

| **Outcomes** | **Probability** | | **OR** | **p-value for test of equivalence to Q1** |
| --- | --- | --- | --- | --- |
|  | **Non-Indigenous** | **Indigenous** |  |  |
| ***Smoking*** |  |  |  |  |
| Quartile 1 | 40 (37-43) | 52 (39-66) | 1.69 (0.95-3.02) | - |
| Quartile 2 | 38 (36-40) | 55 (44-65) | 2.00 (1.28-3.10) | p=0.66 |
| Quartile 3 | 39 (37-42) | 60 (50-70) | 2.40 (1.54-3.72) | p=0.35 |
| Quartile 4 | 41 (38-44) | 73 (66-81) | 4.21 (2.78-6.38) | p=0.01 |
| ***Risky drinking*** |  |  |  |  |
| Quartile 1 | 19 (17-22) | 21 (9-34) | 1.15 (0.54-2.45) | - |
| Quartile 2 | 18 (17-20) | 22 (12-32) | 1.30 (0.70-2.40) | p=0.80 |
| Quartile 3 | 18 (16-21) | 25 (15-34) | 1.47 (0.84-2.54) | p=0.61 |
| Quartile 4 | 21 (18-24) | 40 (28-52) | 2.66 (1.60-4.43) | p=0.07^†^ |
| ***Illicit drug use*** |  |  |  |  |
| Quartile 1 | 43 (40-45) | 46 (32-60) | 1.14 (0.65-2.01) | - |
| Quartile 2 | 38 (36-40) | 44 (34-55) | 1.30 (0.84-2.03) | p=0.72 |
| Quartile 3 | 39 (36-42) | 53 (43-64) | 1.78 (1.16-2.74) | p=0.22 |
| Quartile 4 | 41 (37-44) | 47 (38-57) | 1.31 (0.89-1.94) | p=0.69 |

Notes: Estimated prevalence estimated from marginal effects of multilevel logistic regression models. 95% confidence intervals presented in brackets. Hypothesis testing present p-values for test of equivalence to Q1: † = p < 0.10; *=p < 0.05; ** = p < 0.01; *** = p < 0.001.

### Robustness checks

Table SM6.1 – Logistic regression results for health outcomes under alternate model specifications

|  | **Fair/poor health (OR)** | | | **Poor mental health (OR)** | | | **Disability (OR)** | | |
| --- | --- | --- | --- | --- | --- | --- | --- | --- | --- |
|  | **(1)** | **(2)** | **(3)** | **(1)** | **(2)** | **(3)** | **(1)** | **(2)** | **(3)** |
|  | $\beta$(95%CI) | $\beta$(95%CI) | $\beta$(95%CI) | $\beta$(95%CI) | $\beta$(95%CI) | $\beta$(95%CI) | $\beta$(95%CI) | $\beta$(95%CI) | $\beta$(95%CI) |
| Q1 opposition (ref) | 1.00 | 1.00 | 1.00 | 1.00 | 1.00 | 1.00 | 1.00 | 1.00 | 1.00 |
| Q2 opposition | 1.03  (0.84-1.26) | 1.00  (0.82-1.23) | 1.00 | 0.84  (0.68-1.03) | 0.84  (0.68-1.03) | 1.00 | 0.95  (0.80-1.13) | 0.94  (0.79-1.12) | 1.00 |
| Q3 opposition | 1.09  (0.85-1.40) | 1.12  (0.87-1.43) | 1.00 | 0.80  (0.61-1.05) | 0.78  (0.60-1.02) | 1.00 | 1.18  (0.95-1.46) | 1.18  (0.95-1.45) | 1.00 |
| Q4 opposition | 1.10  (0.83-1.47) | 1.11  (0.83-1.48) | 1.00 | 0.88  (0.65-1.18) | 0.88  (0.65-1.18) | 1.00 | 1.12  (0.89-1.42) | 1.10  (0.87-1.40) | 1.00 |
| Q1 opposition x Indigenous | 1.80  (0.65-4.95) | 1.95  (0.70-5.44) | 1.77  (0.55-5.64) | 1.01  (0.50-2.06) | 1.02  (0.49-2.14) | 0.92  (0.46-1.85) | 1.29  (0.63-2.65) | 1.38  (0.68-2.79) | 1.18  (0.56-2.49) |
| Q2 opposition x Indigenous | 1.44  (0.81-2.55) | 1.59  (0.90-2.80) | 1.39  (0.76-2.52) | 1.69  (1.02-2.78) | 1.71  (1.02-2.84) | 1.67  (0.97-2.88) | 1.53  (0.92-2.57) | 1.57  (0.91-2.72) | 1.64  (0.97-2.77) |
| Q3 opposition x Indigenous | 1.57  (0.93-2.66) | 1.65  (0.97-2.80) | 1.56  (0.89-2.74) | 1.70  (1.04-2.79) | 1.79  (1.07-2.99) | 1.54  (0.89-2.66) | 1.39  (0.88-2.20) | 1.46  (0.92-2.30) | 1.26  (0.80-1.99) |
| Q4 opposition x Indigenous | 2.28  (1.45-3.58) | 2.49  (1.61-3.86) | 2.07  (1.30-3.31) | 2.24  (1.48-3.39) | 2.14  (1.40-3.27) | 2.15  (1.37-3.38) | 1.59  (1.04-2.43) | 1.64  (1.09-2.47) | 1.52  (0.94-2.47) |
| *N* | *14,619* | *14,615* | *14,809* | *14,704* | *14,691* | *14,726* | *15,870* | *15,864* | *16,091* |
| *Pseudo-R2* | *0.07* | *0.09* | *0.14* | 0.02 | 0.04 | 0.09 | 0.1 | 0.12 | 0.21 |

Notes: Exponentiated coefficients. Model (1) shows baseline model. Model (2) specifies age as a continuous variable rather than a categorical variable. Model (3) controls for age group (‘15-39 years,’ ‘40-59 years,’ ‘60 plus years’) and sex (male, female), as in baseline, and additionally controls for labour force status (in labour force, not in labour force), marital status (married or defacto, or single), number children in household, household equivalised income (in quartiles), educational attainment (less than high school, high school or equivalent, university or equivalent), and SA3 fixed effects.

Table SM6.2 – Logistic regression results for healthcare use in past 12 months under alternate model specifications

|  | **Visited any health provider (OR)** | | | **Taken any prescription medications (OR)** | | | **Visited hospital (OR)** | | |
| --- | --- | --- | --- | --- | --- | --- | --- | --- | --- |
|  | **(1)** | **(2)** | **(3)** | **(1)** | **(2)** | **(3)** | **(1)** | **(2)** | **(3)** |
|  | $\beta$(95%CI) | $\beta$(95%CI) | $\beta$(95%CI) | $\beta$(95%CI) | $\beta$(95%CI) | $\beta$(95%CI) | $\beta$(95%CI) | $\beta$(95%CI) | $\beta$(95%CI) |
| Q1 opposition (ref) | 1.00 | 1.00 | 1.00 | 1.00 | 1.00 | 1.00 | 1.00 | 1.00 | 1.00 |
| Q2 opposition | 0.87  (0.74-1.02) | 0.87  (0.74-1.02) | 1.00 | 0.98  (0.84-1.14) | 0.97  (0.83-1.13) | 1.00 | 0.99  (0.85-1.16) | 0.98  (0.84-1.16) | 1.00 |
| Q3 opposition | 0.91  (0.74-1.11) | 0.91  (0.74-1.12) | 1.00 | 1.00  (0.82-1.22) | 1.02  (0.83-1.24) | 1.00 | 1.11  (0.90-1.37) | 1.12  (0.91-1.39) | 1.00 |
| Q4 opposition | 0.88  (0.69-1.11) | 0.88  (0.69-1.11) | 1.00 | 1.01  (0.81-1.27) | 1.00  (0.80-1.26) | 1.00 | 1.23  (0.97-1.56) | 1.22  (0.96-1.55) | 1.00 |
| Q1 opposition x Indigenous | 1.46  (0.80-2.67) | 1.43  (0.77-2.64) | 1.42  (0.75-2.67) | 0.93  (0.53-1.65) | 0.99  (0.56-1.76) | 0.95  (0.53-1.71) | 1.70  (0.86-3.36) | 1.77  (0.90-3.50) | 1.70  (0.82-3.50) |
| Q2 opposition x Indigenous | 0.75  (0.48-1.18) | 0.79  (0.50-1.24) | 0.74  (0.46-1.18) | 0.97  (0.61-1.56) | 1.04  (0.67-1.63) | 1.02  (0.63-1.65) | 1.61  (0.99-2.61) | 1.66  (1.01-2.71) | 1.42  (0.86-2.36) |
| Q3 opposition x Indigenous | 0.73  (0.47-1.13) | 0.70 (0.45-1.08) | 0.89  (0.57-1.38) | 0.93  (0.59-1.45) | 0.96  (0.61-1.50) | 1.01  (0.64-1.59) | 1.33  (0.79-2.23) | 1.41  (0.84-2.37) | 1.45  (0.86-2.43) |
| Q4 opposition x Indigenous | 0.52  (0.36-0.75) | 0.52  (0.36-0.75) | 0.70  (0.49-1.01) | 1.18  (0.83-1.67) | 1.22  (0.86-1.74) | 1.35  (0.93-1.96) | 1.14  (0.76-1.71) | 1.21  (0.79-1.83) | 1.19  (0.76-1.86) |
| *N* | *15,917* | *15,911* | *16,231* | *15,920* | *15,849* | *16,236* | *15,921* | *15,917* | *16,237* |
| *Pseudo-R2* | *0.05* | *0.06* | *0.1* | *0.12* | *0.13* | *0.15* | *0.03* | *0.04* | *0.06* |

Notes: Exponentiated coefficients. Model (1) shows baseline model. Model (2) specifies age as a continuous variable rather than a categorical variable. Model (3) controls for age group (‘15-39 years,’ ‘40-59 years,’ ‘60 plus years’) and sex (male, female), as in baseline, and additionally controls for labour force status (in labour force, not in labour force), marital status (married or defacto, or single), number children in household, household equivalised income (in quartiles), educational attainment (less than high school, high school or equivalent, university or equivalent), and SA3 fixed effects.

Table SM6.3 – Logistic regression results for risk-taking behaviours under alternate model specifications

|  | **Smoker/ex-smoker (OR)** | | | **Risky drinking (OR)** | | | **Any illicit drug use (OR)** | | |
| --- | --- | --- | --- | --- | --- | --- | --- | --- | --- |
|  | **(1)** | **(2)** | **(3)** | **(1)** | **(2)** | **(3)** | **(1)** | **(2)** | **(3)** |
|  | $\beta$(95%CI) | $\beta$(95%CI) | $\beta$(95%CI) | $\beta$(95%CI) | $\beta$(95%CI) | $\beta$(95%CI) | $\beta$(95%CI) | $\beta$(95%CI) | $\beta$(95%CI) |
| Q1 opposition (ref) | 1.00 | 1.00 | 1.00 | 1.00 | 1.00 | 1.00 | 1.00 | 1.00 | 1.00 |
| Q2 opposition | 0.93  (0.80-1.07) | 0.91  (0.79-1.06) | 1.00 | 0.93 (0.77-1.12) | 0.95  (0.78-1.15) | 1.00 | 0.83  (0.72-0.95) | 0.82  (0.71-0.94) | 1.00 |
| Q3 opposition | 0.97  (0.81-1.17) | 0.99  (0.82-1.19) | 1.00 | 0.94  (0.73-1.22) | 0.97  (0.75-1.24) | 1.00 | 0.86  (0.72-1.04) | 0.86  (0.72-1.03) | 1.00 |
| Q4 opposition | 1.04  (0.84-1.29) | 1.04  (0.84-1.29) | 1.00 | 1.11  (0.82-1.49) | 1.14  (0.85-1.54) | 1.00 | 0.92  (0.75-1.13) | 0.91  (0.74-1.12) | 1.00 |
| Q1 opposition x Indigenous | 1.69  (0.95-3.02) | 1.78  (0.97-3.26) | 1.53  (0.82-2.87) | 1.15  (0.54-2.45) | 1.07  (0.49-2.32) | 1.02  (0.49-2.12) | 1.14  (0.65-2.01) | 1.17  (0.65-2.09) | 0.98  (0.50-1.90) |
| Q2 opposition x Indigenous | 2.00  (1.28-3.10) | 2.08  (1.33-3.27) | 1.71  (1.06-2.76) | 1.30  (0.70-2.40) | 1.31  (0.71-2.42) | 1.22  (0.64-2.31) | 1.30  (0.84-2.03) | 1.31  (0.84-2.03) | 1.18  (0.75-1.86) |
| Q3 opposition x Indigenous | 2.40  (1.54-3.72) | 2.74  (1.74-4.32) | 1.97  (1.26-3.06) | 1.46  (0.84-2.54) | 1.41  (0.81-2.48) | 1.26  (0.69-2.30) | 1.78  (1.16-2.74) | 1.83  (1.21-2.76) | 1.77  (1.17-2.69) |
| Q4 opposition x Indigenous | 4.21  (2.78-6.38) | 5.05  (3.23-7.88) | 3.62  (2.35-5.59) | 2.66  (1.60-4.43) | 2.72  (1.65-4.47) | 2.40  (1.41-4.06) | 1.31  (0.89-1.94) | 1.33  (0.90-1.97) | 1.12  (0.74-1.68) |
| *N* | *14,658* | *14,652* | *14,942* | *11,578* | *11,493* | *11,765* | *14,585* | *14,581* | *14,850* |
| *Pseudo-R2* | *0.04* | *0.07* | *0.09* | *0.03* | *0.05* | *0.10* | *0.01* | *0.01* | *0.04* |

Notes: Exponentiated coefficients. Model (1) shows baseline model. Model (2) specifies age as a continuous variable rather than a categorical variable. Model (3) controls for age group (‘15-39 years,’ ‘40-59 years,’ ‘60 plus years’) and sex (male, female), as in baseline, and additionally controls for labour force status (in labour force, not in labour force), marital status (married or defacto, or single), number children in household, household equivalised income (in quartiles), educational attainment (less than high school, high school or equivalent, university or equivalent), and SA3 fixed effects.

# References

ABS. 2022. *Person Level Integrated Data Asset (PLIDA)* [Online]. Available: <https://www.abs.gov.au/about/data-services/data-integration/integrated-data/person-level-integrated-data-asset-plida> [Accessed 1 January 2024].

HARFIELD, S., PEARSON, O., MOREY, K., KITE, E., CANUTO, K., GLOVER, K., GOMERSALL, J. S., CARTER, D., DAVY, C. & AROMATARIS, E. 2020. Assessing the quality of health research from an Indigenous perspective: the Aboriginal and Torres Strait Islander quality appraisal tool. *BMC medical research methodology,* 20**,** 1-9.

SAXBY, K., DICKINSON, H., PETRIE, D., KAVANAGH, A. & AITKEN, Z. 2023. The impact of employment on mental healthcare use among people with disability: distinguishing between part-and full-time employment. *Scandinavian Journal of Work, Environment and Health,* 49**,** 598-609.
